# Nurse-midwifery education through graduate programs to provide a sufficient number of high quality nurse-midwives

**DOI:** 10.3352/jeehp.2006.3.5

**Published:** 2006-12-05

**Authors:** Kyung Hye Lee

**Affiliations:** College of Nursing science, Ewha Womans University, Seoul, Korea.

**Keywords:** Midwifery, Education, Medical Laws, Curriculum, Graduate program

## Abstract

There is a decrease in the number of new midwives, resulting from the shutdown of midwifery education program in hospitals due to a decrease in birthrate in the Republic of Korea. To solve this problem, the current medical laws on midwifery education system in Korea should be revised; nurse-midwifery specialist programs must be established in educational institutes with nursing programs. To support this argument, the midwifery education programs of America, Europe, Australia, and Japan have been discussed, and a nurse-midwifery specialist curriculum at the master's level, based on the nurse-practitioner system of Korea, has been suggested. Since this assertion is very important and urgent for solving the future population problem of Korea and providing health care for women and children, it should be realized into action immediately.

## INTRODUCTION

The midwife is a health professional with a nursing license, who has also obtained a midwife license after receiving one year of midwifery education and passing a national licensing examination. According to Article 3 of the Enforcement Ordinance of Medical Code, midwifery education must be performed in "obstetrics/gynecology and pediatric hospitals with the average of a hundred or more childbirths per month." Therefore, most directors of hospitals submit a request to the Ministry of Health and Welfare and perform midwifery education programs? In order to become a midwife, one must receive a yearlong education in a hospital that provides this midwifery program, pass the national examination and receive a license from the Ministry of Health and Welfare. Up until 1991, more than 200 students received midwifery education and applied for the national licensing examination (240 in 1990, 220 in 1991). However, due to the sudden decrease in birth rate (1.16) [1], the childbirth number in many university hospitals has lowered, and midwifery programs had to be shut down. Therefore the number of midwifery students has sharply decreased, from 240 in 1991 to only 65 in 2004. Such tendency makes significant problem for the supply of midwives. The current childbirth tendency avoids unnecessary Caesarian sections or medical operations in hospital delivery, preferring family-centered natural delivery instead. In accordance with this, the childbirth rate in some midwifery clinics is on the increase each month (28 births in March 2005 as compared with 36 in December, provided by Ilsin Midwifery clinic). Considering that the expectant mothers coming to midwifery clinics are highly-educated upper-class women, it can be said that, when pregnant women choose the way and place of delivery, more and more women prefer to deliver naturally in midwifery clinics than in hospitals. In order to meet the requirements of the women who want to give natural childbirths, the supply of midwives with professional knowledge and practical skills is most important. In developed countries such as the United States, universities with medical or nursing schools have midwifery education options such as master's (M.A.) programs, and the curriculum of each institute is evaluated and approved by the standards of the America College of Nurse-Midwifery in order to improve and maintain the quality of midwifery education. According to globalization, the opening of health services requires medical professionals, including midwives, to provide service and share knowledge and skills for the benefit of the health of the entire world as well as the nation [2]. In order to satisfy this requirement, a graduate school-level of midwifery education is necessary. Midwifery education in the graduate school curriculum will not only improve the quality of midwives but also expand the hospital-exclusive midwifery education to universities and increase the number of midwives, thereby efficiently providing midwives. Therefore, this research strongly asserts that nurse-midwifery education program on a M.A. level must be practiced in universities with nursing departments and explains its necessity by discussing the midwifery curriculum of Korea and other countries and suggesting the curriculum of nurse-midwifery education in graduate programs.

## MIDWIFERY EDUCATION CURRICULUNM IN FOREIGN COUNTRIES

### Midwifery Education in the United States

In the United States, a nurse with a nursing license can become a certified nurse midwife after completing M.A. curriculum for two or more years with options of midwifery classes and passing the license examination operated by the ACNM (America College of Nurse-Midwives) DOA (Division of Accreditation). Midwifery programs are available in schools with medical or nursing schools, and the curriculum and education periods differ among institutions. In order to improve and maintain the quality of American midwifery education, the different curriculums of institutions are evaluated and approved according to the standards of the ACNM. The ACNM presents the core abilities for midwifery work and requires curriculums to be based on it. This core ability is revised and modified every five years to comply with the changing demands of society. Therefore, the midwifery curriculum of American universities offers 40 to 50credits to meet the ACNM standards. Students can choose 2-year, 2-and-half-year, or 3-year programs depending on their status as full- or half-time students. The nurse-midwifery curriculum of United States is the certified nurse midwife program, and thesis writing is or is not mandatory depending on the universities. A thesis is required for entering the doctorate program later on but not when students aim to enter general practice; in this case, practice is emphasized in place of thesis writing. The following is an introduction to the curriculum of University of Illinois, in which a thesis is mandatory. The educational purpose of the midwifery education program at the University of Illinois at Chicago, College of Nursing, is as follows (http://www.uic.edu/nursing/):

#### Clinical Practice Ability

1. Satisfies the wishes of pregnant women and their families as humans in whole.
2. Continues health care for pregnant women.
3. Carries out practical service with safe performance ability.
4. Does not intervene unnecessarily in normal processes.
5. Performs health education throughout the whole life of a woman.

#### Concerning the Object

1. To emphasize human dignity and cultural diversity.
2. To appreciate self-decisions.
3. To provide correct, easy-to-understand information.
4. To encourage active participation of the pregnant women and her family?

#### Cooperation with Other Medical Personnel

1. Cooperates with other medical teams.
2. Learns new knowledge and develops professional leadership.
3. Uses knowledge of humans, environment, and health.
4. Evaluates the efficiency of midwifery work.
5. Encourages research for developing knowledge.

### 

The curriculum to achieve these purposes is as Table 1. The graduate school curriculum of midwifery education in the College of Nursing at the University of Chicago emphasizes not only the nursing of pregnant women and newborns but also female health care throughout a woman's life span. The 2-year program emphasizes theory in the first year and practice in the second year. For a degree, students are required to take research project, statistics, and theory, etc. as common subjects.

The Shenandoah University (http://www.su.edu) midwifery education program consists of a 2-year midwifery education after achieving a nursing license. Instead of thesis writing, practice is more heavily emphasized. Students take common subjects in the first year and concentrate on studying and practicing midwife-related subjects in the second. Each semester, theory and practice are carried out in parallel. In the first year, students take gynecology, primary health care, health care of pregnant women, practice; in the second year, students take, labor & delivery, postpartum, health care of newborn, practice, law, management, elective. In total, students take 44 credits: 22 in their major, and 22 in common and related subjects.

### Midwifery Education in Europe

Midwifery in Europe is more actively practiced than in America. Curriculums vary among countries, and the United Kingdom, Netherlands, Germany and Sweden each has a slightly different educational system and curriculum [3]. In the United Kingdoms, there is the Certified Nurse Midwife (CNM), who becomes a midwife after completing one and a half years of midwifery education programs, and the Certified Midwife (CM), who completes a three-year program. The curriculums are offered in colleges of nursing, medical schools, and hospitals. In Germany, one who finishes a three-year midwifery program becomes a CM. In the Netherlands, colleges offer three-year midwifery education. Sweden's universities perform midwifery education as a 4-year BS program. The curriculum of Karolinska Institutet (formerly Stockholm University College of Health Science) in Sweden is as follows [4]:
  
Elementary programs                                        20 credits
Advanced programs for pregnant women and family planning   10 credits
Advanced programs for childbirth and family nursing        20 credits
Advanced programs for women's health care                  10 credits
Total                                                      60 credits
  
  

In each program, nursing science programs about women's reproduction health, medicine and natural science are included. In medical and natural science, anatomy, physiology, endocrinology, uterus diagnostics, neonatal studies, obstetrical disease, pregnancy complications, clinical sexology, family planning, abnormal delivery, microbiology, infectious disease, pharmacology, the scientific method, and gynecology are required. As to sociology and behavioral sciences, community nursing, psychology, sociology, general sexology, law, and the scientific research method. Although the Swedish curriculum is not a graduate program, the four-year undergraduate program includes extensive knowledge in natural science, sociology and behavioral science. The midwifery education program of Sweden consists of the subjects necessary to understand humans holistically.

### Midwifery Education in Australia

The nursing colleges of Australia divide into the Department of Nursing and Department of Midwifery. Each department offers a bachelors degree, postgraduate diploma program, master's program, and doctorate program [5]. Only nurses who have received a bachelor's degree or the equivalent level of education and have more than one year of clinical experiment can enter the programs. The four semesters consist of the regular 15 months and then 12 part-time months. Required credits are a total of 80 units. For example, the midwifery education subjects of diploma program at the University of Newcastle requires the general common programs of college studies, clinical practice, research methods, midwifery practices 1, 2, and 3, recent midwifery, issues in midwifery practice, mothers, and families as required major programs. Students must choose one program from theoretical frame of midwifery practice, co-operational practice in mother-child health, and teaching-learning in midwifery situations to finish their degree. For the master's degree, a research proposal and thesis writing is included in the requirements program.

### Midwifery Education in Japan

Midwifery education in Japan is offered at Colleges of Nursing and consists of a 4-year program. Students who wish to become midwives choose the midwifery program in their third year and practice midwifery. After graduation, students can earn both a nursing license and a nurse midwife license through a national exam. For example, the University of Hiroshima requires students to take Introduction to Midwifery, and Midwifery Studies 1 and 2 in their third year and spend 225 hours practicing midwifery in the fourth year. The advantage of the midwifery curriculum of Japan is that all programs are 4-year bachelor's degree programs [6].

## THE REALITY OF MIDWIFERY EDUCATION in KOREA

As for curriculum, Article 4 of Ministry of Health and Welfare Order states that "midwifery practice program should be 40 hours a week including program studies and practices, and the curriculum is stated in the attached Table 2." Currently, the number of childbirths in Korea does not exceed 100 per month in most university hospitals. This number is not sufficient even to train obstetrics & gynecology doctors and midwifery students. Moreover, Caesarian sections account for more than 38.6% of childbirths, decreasing the number of normal deliveries even more [7].

In this situation, there is a lack of cases for midwives-in-training to practice midwifery. As a result, midwife education programs in hospitals are at risk of being closed down.?

The suspension or closure of midwifery education may bring great crisis to the mother-child health policy and supply of midwives in the future. The lowering birthrate indicates a decrease in important human resources that results in the decline of national competitiveness. Therefore the Government is carrying out childbirth encouragement policies and education that encourages natural delivery instead of Caesarian section. Women want to decide for themselves where and how they will give birth to their children. They want to give natural childbirths in familial settings and experience the mystery and wonder of birth. Consequently, women who want to give childbirth in midwifery clinics are increasing, rising from 43 births in January to 115 in March [8]. Most women who prefer childbirth in midwifery clinics are professionals with high self-esteem or are highly educated and economically well off. This is a completely different tendency from the past, when poor, uneducated women went to midwifery clinics because they couldn't afford hospitals.

However, it is difficult to meet the demands of pregnant women who want to deliver in midwifery clinics if the numbers of midwifery clinics and the quality of midwives are low. Therefore, professional midwives with holistic nurse-midwifery competency must be produced to carry out a high quality of midwifery work.

In order to produce qualified midwives, education at the graduate-school level is necessary. However, the current medical law of Korea prohibits midwifery education in educational institutes (college/department of nursing). Thus, current medical law must be revised first of all.

## SUGGESTION OF REVISION OF MEDICAL LAWS AND CURRICULUM FOR NURSE-MIDWIFERY SPECIALISTS

### Revision of Medical Laws

The laws that need to be revised in order to enable nurse-midwifery education in graduate school are: medical law article 6, related to the license of nurse-midwives; medical law enforcement regulation article 3, related to medical institutions, educational institutes, and the number of students related to midwifery education; and order of Ministry of Health and Welfare article 4, "containing the subjects and practicum of midwifery practice program explained in Table 3".

#### Revision of Medical Law Article 6

"Nurse-midwife who possess nursing license and have finished one-year midwifery practice program in medical institutions approved by the Minister of Health and Welfare." This article should be altered as follows: "Nurse-midwife who possess nursing license and have finished a master's program in nurse-midwifery curriculum at a nursing education institute."

Nurse-midwives perform family-centered, professional midwifery and are not only involved in the delivery process but also the whole lives of women. Nurse-midwives also act as leaders in midwifery education, practice, and policy. Those who wish to improve themselves more academically can connect their studies to doctorate programs, thereby contributing to the theoretical improvements of midwifery. This enables the exchange of knowledge and techniques between midwives around the world.

#### Revision of the Order of Ministry of Health and Welfare Issue 712

Currently in Korea, the Order of Ministry of Health and Welfare Issue 712, regulations of midwifery practice article 4, clarifies that a midwifery practice program must consist of program studies and practice of 40 hours per week, requiring a total of 200 hours of program studies, 1,720 hours of practice, and over 20 direct birth assistant. Because this article must be adjusted for a master's-degree level of nurse-midwifery curriculum, we suggest the following adjustments.

### Curriculum for Nurse-midwifery Specialists

Nurse-midwife education curriculums have been designed based on 1) the midwifery regulation of World Health Organization (WHO) [9], 2) a core competency of midwives defined by the International Council of Midwife [10] and ACNM [11], 3) the midwifery curriculum of foreign countries (U.S.A., Australia, Sweden), 4) an analysis of the duties of midwives in Korea [12], 5) a validity study on the subjects of midwife national examination [13], and 6) a nurse practitioner curriculum of Ministry of Health and Welfare.

#### Philosophy of Education

The mission of nurse-midwifery specialist programs is to educate nurse-midwives who can provide safe, comprehensive, evidence-based health care to women. Nurse-midwives who have completed this program will work as midwives in both hospitals and the community and be primary health care specialists in public health centers. This proposed masters in midwifery program, by providing comprehensive health care and appropriate health education throughout a woman's life span, promotes health maintenance and enhancement and prevention of diseases. Primary health care, reproductive health care, health care during pregnancy, labor & delivery, postpartum women, contraception & family planning, newborn & infant health, and family health care classes are offered. Nurse-midwives provide health care based on knowledge of health science that focuses on midwifery work and on women and family-centered practice. Nurse-midwives respect women's decisions and different cultures and lifestyles, as well as the individuality and spiritual dignity of every woman.

#### Purpose of Education

Nurse-midwifery specialist programs provide nurse-midwives with the following abilities:

- professional knowledge as clinical nurse-midwives
- the ability to provide not only scientific knowledge on reproductive health and specific, comprehensive knowledge related to it, but also evidence-based health care
- the ability to consider, plan, practice, and evaluate objects through the nursing process in diverse practice situations
- the knowledge, attitude, and technique to have problem-solving capacity in emergencies
- creativity and the ability to use oneself as an instrument?
- the ability to cooperate with other health care personnel in diverse health care systems
- the ability to contribute to society by realizing their rights and duties as professionals, clinical practice researchers, educators, and scholars

#### Curriculum

This curriculum is based on the Nurse-Practitioner program approved by the Ministry of Health and Welfare, which consists of 13 credits of common theory subjects, 10 mandatory credits, and 10 practice credits (total 33 credits).

#### Educational Evaluation

- The evaluation of nurse-midwives is based on the standards of nurse-practitioners provided by the Ministry of Health and Welfare.
- The curriculum of nurse-midwifery education is evaluated every 5 years by the Midwifery Education Evaluation Committee in Korea Midwife Association. The details to be evaluated are the educational philosophy and purpose, the qualification of faculties and preceptors, facilities (for study and practice), present conditions of study and practice standards, status of educational institutes and practice centers, and scholarship offers, etc.
- The ratio of successful applicants for national examination.
- The ratio and conditions of graduated students' employment.

## CONCLUSION

This paper explained the necessity of revising the current medical laws to increase of numbers of midwife and improve the quality of Korean midwives and suggested specific revisions of medical laws and graduate level curriculum for nurse-midwifery education. These suggestions must be realized in order to provide a sufficient number of high quality midwives. Not only that, it is necessary to realize the childbirth encouragement policy of Ministry of Health and Welfare and the worldwide health policy of WHO, which promotes "less Caesarian section delivery, more natural childbirth." As this proposal is very urgent and important for Korean health policy, it should be realized into action immediately.

## Figures and Tables

**Table 1:**
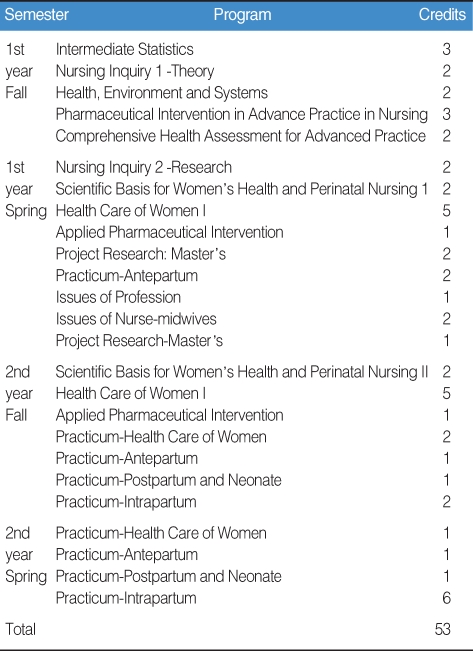
Curriculum for the midwifery education program at the University of Illinois at Chicago, College of Nursing

**Table 2:**
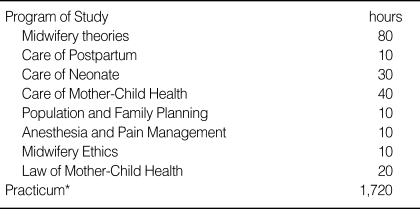
Midwifery Practice Program

^*^Practicum can be done in midwifery clinics as well as hospitals and public health centers. Through the practice, one must act as a delivery attendance for more than 20 childbirths. In such cases, reports must be submitted to the chief of the practice institute.

**Table 3:**
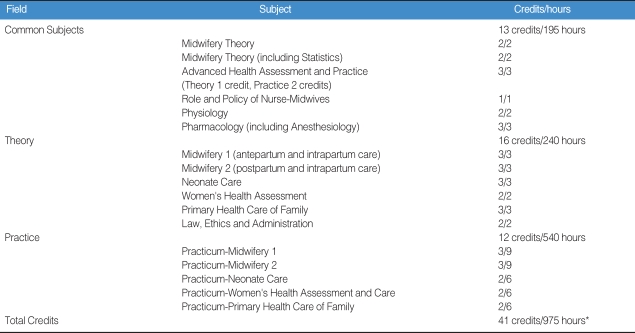
Nurse-midwifery Specialist Curriculum

^*^33 out of 41credits are required.
